# Data of expression and purification of recombinant Taq DNA polymerase

**DOI:** 10.1016/j.dib.2016.08.032

**Published:** 2016-08-20

**Authors:** Na Fang, Niannian Zhong, Yueyang Yang, Yujian Guo, Shaoping Ji

**Affiliations:** Medical School of Henan University, Kaifeng, Henan, China

**Keywords:** Taq DNA polymerase, pET-28b, Expression, Purification

## Abstract

Polymerase chain reaction (PCR) technique is widely used in many experimental conditions, and Taq DNA polymerase is critical in PCR process. In this article, the Taq DNA polymerase expression plasmid is reconstructed and the protein product is obtained by rapid purification, (*“Rapid purification of high-activity Taq DNA polymerase”* (Pluthero, 1993 [1]), *“Single-step purification of a thermostable DNA polymerase expressed in Escherichia coli”* (Desai and Pfaffle, 1995 [2])). Here we present the production data from protein expression and provide the analysis results of the production from two different vectors. Meanwhile, the purification data is also provided to show the purity of the protein product.

**Specifications Table**

**Table:** 

Subject area	*Molecular biology*
More specific subject area	*Protein expression and purification*
Type of data	*Figures*
How data was acquired	*Sodium dodecyl sulfate polyacrylamide gel electrophoresis (SDS-PAGE)*
Data format	*Raw, analyzed*
Experimental factors	*Expression vectors: pTTQ18, pET-28b. Inducer concentration, time and temperature during protein expression.*
Experimental features	*Production levels of Taq DNA polymerase, protein purity.*
Data source location	*Kaifeng, Henan, China*
Data accessibility	*The data is with this article.*

**Value of the data**

• The data showing the ability of the pET system to improve protein expression is useful for the quantification of specific protein levels in a great number of samples.
• The Taq DNA polymerase gene is subcloned from pTTQ18 vector into pET-28b vector and the data shows a great increase of the level of the protein expression.
• The data shows that the purified protein product contains very few hetero-proteins, indicating that its purity and/or activity level would be high and in favor of the PCR reaction.

## 1 Data

**Fig. 1:**
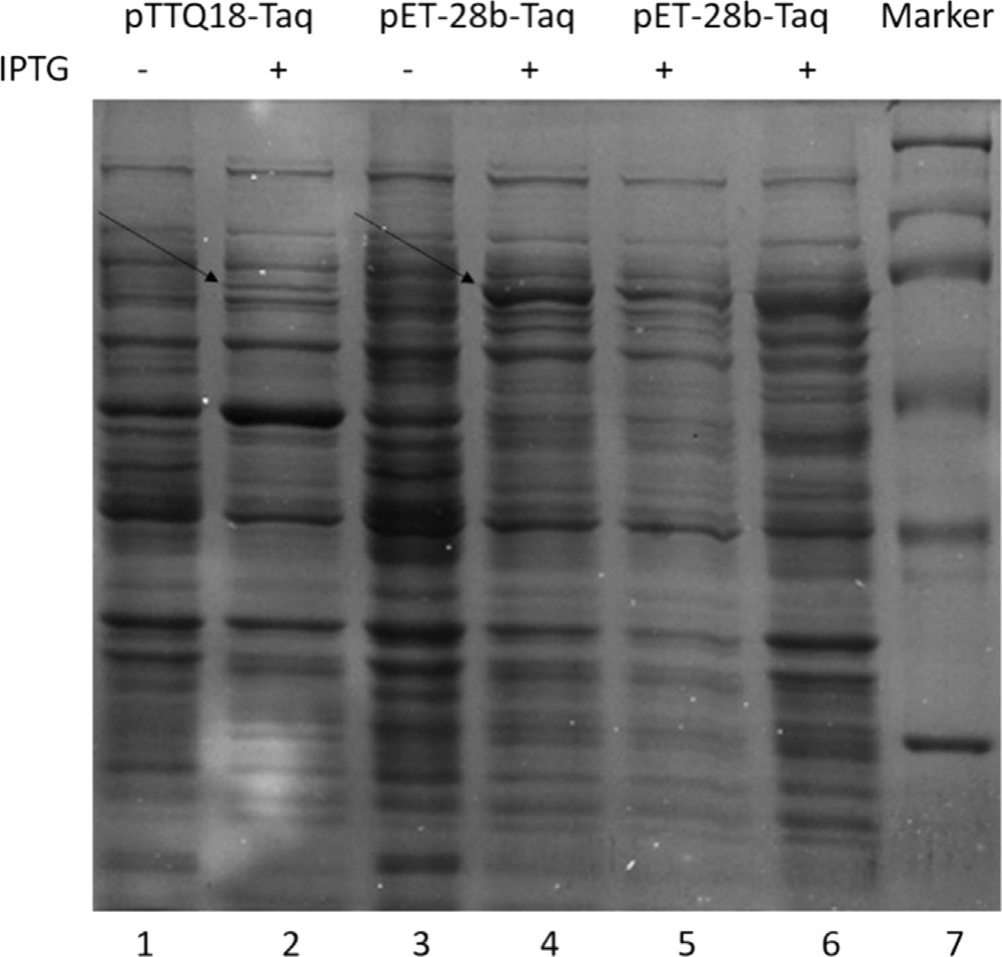
10% SDS-PAGE analysis of Taq DNA polymerase expressed in E.coli. Lane 1: proteins expression of the recombinant plasmid pTTQ18 without IPTG induction. Lane 2: proteins expression of the recombinant plasmid pTTQ18 with IPTG induction. Lane 3: proteins expression of the recombinant plasmid pET-28b without IPTG induction. Lane 4: proteins expression of the recombinant plasmid pET-28b with IPTG induction. Lane 5: supernatant of induced pET-28b recombinant bacteria. Lane 6: precipitation of induced pET-28b recombinant bacteria. Lane 7: protein marker.

**Fig. 2:**
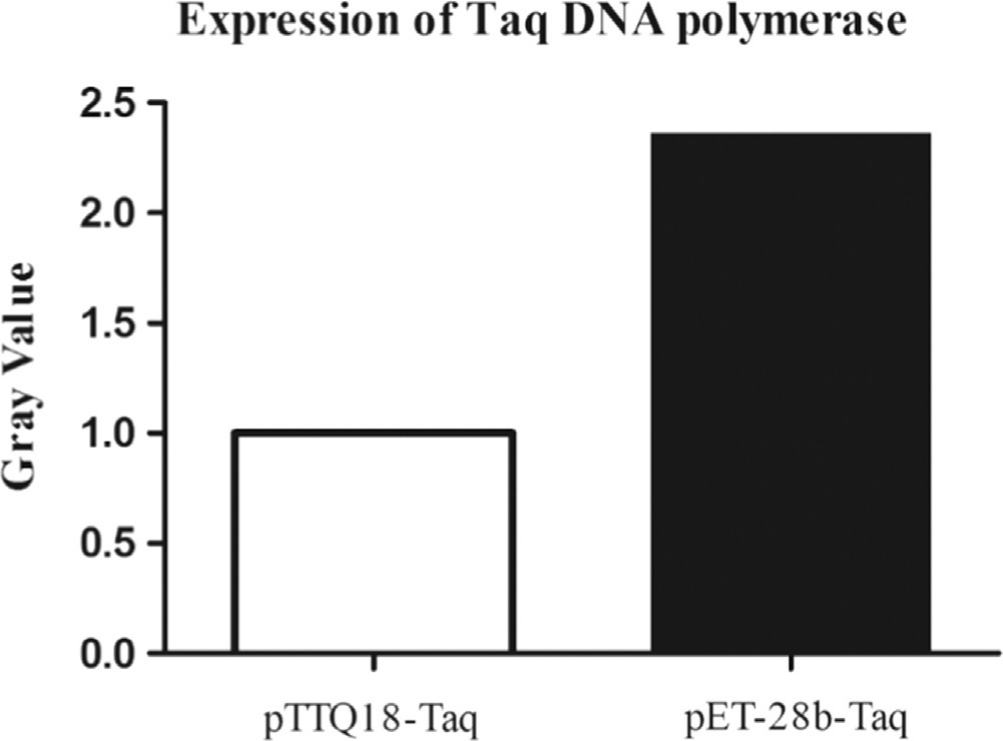
Gray scale value analysis of the expression of Taq DNA polymerase.

Fig. 1 shows the SDS-PAGE analysis of the Taq DNA polymerase expression in pET-28b, and Fig. 2 shows the gray scale value analysis result of the recombinant Taq DNA polymerase. A considerable increase of the protein expression can be seen.

**Fig. 3:**
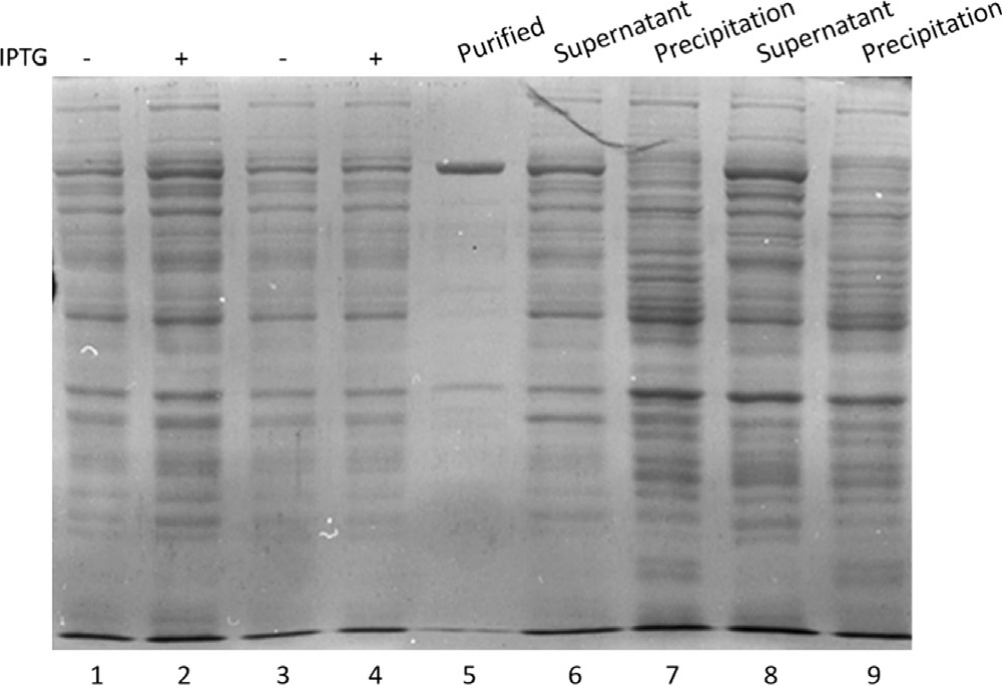
10% SDS-PAGE analysis of the purification of Taq DNA polymerase from pET-28b recombinant. Lane 1 and 3: protein expression before induced. Lane 2 and 4: protein expression after induced. Lane 5: purified protein. Lane 6 and 8: supernatant of lane 2 and 4 respectively. Lane 7 and 9: precipitation of lane 2 and 4 respectively.

Fig. 3 is the SDS-PAGE analysis of the Taq DNA polymerase purification from pET-28b recombinant, which shows the purity of the purified protein product.

## 2 Experimental design, materials and methods

### 2.1 Plasmid construction

The polymerase gene was amplified by PCR from the template plasmid, which contains the target gene in pTTQ18 vector. The up- and down-stream DNA primers were 5′-CATATGCGGGGGATGCTGCCCCTCTT-3′ and 5′-GAATTCTCACTCCTTGGCGGAGAGCCAGTC-3′ with restriction sites of Nde I and EcoR I respectively. The 2508 bp target gene fragment of PCR was inserted into pGEM T-Easy plasmid using T/A cloning. The target DNA was verified by sequencing. Both the recombinant plasmid and pET-28b plasmid were then digested by Nde I and EcoR I, respectively. Subsequently, the 2508 bp fragment was ligated into pET-28b plasmid by T_4_ DNA ligase.

### 2.2 Protein expression

The pTTQ18 and pET-28b recombinant plasmid were transformed into competent *E. coli* bacteria BL21(DE3) and BL21 respectively for expression [3]. Both of them were processed by the same expression procedure.

1 ml of the over-night cultured medium was transferred into 200 ml of LB broth (1:200) to grow. Isopropyl-b-D-thiogalactopyranoside (IPTG, final concentration: 1 mM) was then added into growth medium when the OD_600_ of the culture was between 0.3 and 0.6. After being cultured for additional 4–6 h, cells were harvested by centrifugation. The supernatant was discarded and 20 ml of PBS(137 mM NaCl, 2.7 mM KCl, 10 mM Na_2_HPO_4_, 2 mM KH_2_PO_4_, pH7.8) was used to re-suspend the cell pellet. 50 μL of the suspension was taken out for expression identification, and then the remained cells were washed by centrifugation. The suspension was analyzed by SDS-polyacrylamide gel electrophoresis(10%) [4]. The pellets of rest cells were harvested and temporarily stored at −80 °C.

### 2.3 Protein purification

The frozen cell pellets were re-suspended in 10 ml of buffer A [50 mM Tris–HCl (pH 7.9), 50 mM glucose, 1 mM EDTA, 4 mg/mL of lysozyme (fresh added)] for 15 minutes at room temperature. Then 10 ml buffer B [10 mM Tris–HCl (pH7.9), 50 mM KCl, 1 mM EDTA, 0.5% Tween 20, 0.5% Nonidet-P40, 1 mM PMSF (fresh added)] was added, followed by an incubation at 75 °C for 1.0 h with tube inversion every 10 minutes [1], [2].

The samples were centrifuged and the supernatant was transferred into a new tube. Every 100 ml volume of the samples was added 30 g powered (NH_4_)_2_SO_4_. The samples were centrifuged and the supernatant was discarded. The both precipitated and floated protein was harvested and 4 ml PBS was added to dissolve the protein. The samples were dialyzed in buffer C[20 mM Tris–HCl (pH 8.0 at 25 °C), 100 mM KCl, 0.1 mM EDTA, 1 mM DTT, 0.5% Tween 20 and 0.5% Nonidet-P40, 33.3% glycerol (add after dialysis)] with 3 times of replacement of the buffer every 4 h. Glycerol was added to 33.3% in the final polymerase product which would be stored at −80 °C. 20 μL of the protein was used for a SDS-PAGE analysis for purity.
